# When Is Postpartum Haemorrhage Treatment Initiated? A Nested Observational Study Within the E‐MOTIVE Trial

**DOI:** 10.1111/1471-0528.18293

**Published:** 2025-08-04

**Authors:** Kristie‐Marie Mammoliti, Fernando Althabe, Christina Easter, Adam Devall, James Martin, Adeosun Love Funmi, Rahmatu Yusuf, Fatima Abubakar, Lolade Christiana Arigbede, JimKelly Mugambi, Polycarp Oyoo, Masumbuko Sambusa, Akwinata Banda, Fawzia Samuels, Sara Willemse, Sibongile Doris Khambule, Hilal Mukhtar Shu'aib, Aminu Ado Wakili, Jenipher Okore, Ard Mwampashi, Mandisa Singata‐Madliki, Edna Arends, Elani Muller, Hadiza Galadanci, Zahida Qureshi, Fadhlun Alwy Al‐Beity, G. Justus Hofmeyr, Sue Fawcus, Neil Moran, George Gwako, Alfred Osoti, Ioannis Gallos, Arri Coomarasamy

**Affiliations:** ^1^ College of Medicine and Health University of Birmingham Birmingham UK; ^2^ UNDP‐UNFPA‐UNICEF‐WHO‐World Bank Special Programme of Research, Development and Research Training in Human Reproduction (HRP), Department of Sexual and Reproductive Health and Research World Health Organization Geneva Switzerland; ^3^ African Center of Excellence for Population Health and Policy, College of Health Sciences Bayero University Kano Nigeria; ^4^ Department of Obstetrics and Gynecology University of Nairobi Nairobi Kenya; ^5^ Department of Obstetrics and Gynecology Muhimbili University of Health and Allied Sciences Dar es Salaam Tanzania; ^6^ Department of Obstetrics and Gynaecology University of Cape Town Cape Town South Africa; ^7^ Effective Care Research Unit University of the Witwatersrand Johannesburg South Africa; ^8^ Walter Sisulu University Mthatha South Africa; ^9^ Department of Obstetrics and Gynecology University of Botswana Gaborone Botswana; ^10^ KwaZulu‐Natal Department of Health Pietermaritzburg South Africa; ^11^ Department of Obstetrics and Gynaecology, Nelson R Mandela School of Medicine University of KwaZulu‐Natal Durban South Africa

**Keywords:** MOTIVE bundle, postpartum haemorrhage, PPH, PPH treatment, PPH treatment bundle, sub‐Saharan Africa

## Abstract

**Objective:**

To compare the frequency and timing of postpartum haemorrhage (PPH) treatment initiation between hospitals implementing the MOTIVE treatment bundle (which consisted of uterine Massage, Oxytocic drugs, Tranexamic acid, IntraVenous fluids and Examination) and those following usual care.

**Design:**

Nested prospective observational study.

**Setting:**

Hospitals in Nigeria, Kenya, Tanzania and South Africa participating in the E‐MOTIVE trial.

**Population or Sample:**

Healthcare workers treating PPH.

**Methods:**

Between June and December 2022, we observed healthcare workers for 1–2 weeks in 39 E‐MOTIVE and 39 usual care hospitals across Nigeria, Kenya, Tanzania, and South Africa managing vaginal birth and treating PPH. We descriptively compared the frequency and timing from PPH detection to treatment initiation of individual treatments and the MOTIVE bundle, between E‐MOTIVE care and usual care.

**Results:**

Among 2578 observations in E‐MOTIVE care hospitals, 295 (11%) PPHs were treated, and among 2834 observations in usual care hospitals, 219 (8%) PPHs were treated. In E‐MOTIVE care hospitals, 97% (286/295) of women with PPH received the MOTIVE bundle, compared to 36% (79/219) in usual care. Median initiation times for the first component were similar (0 vs. 1 min), but E‐MOTIVE care hospitals achieved faster initiation of all components (13 min, IQR 6–18) compared to usual care (18 min, IQR 10–25). In total, 79% (233/295) of women in E‐MOTIVE care had all components initiated within 20 min, compared to 22% (48/219) in usual care.

**Conclusions:**

Timely and comprehensive management of PPH using the MOTIVE bundle, particularly initiating all components within 15–20 min, was commonly observed in the E‐MOTIVE care hospitals. Scaling up E‐MOTIVE care should emphasise timely bundle initiation to strengthen PPH treatment and improve maternal health outcomes in low‐and‐middle‐income countries.

## 1 Introduction

The world sees the tragic loss of a mother every 7 min due to postpartum haemorrhage (PPH) [1], which constitutes 27% of all maternal deaths, making it the leading cause of maternal mortality globally [2]. This issue disproportionately affects women in low‐ and middle‐income countries (LMICs) [3]. For many of these women, the challenges do not end at the detection of PPH, which is well documented to be poor [4]; significant delays in PPH treatment and inconsistencies in the initiation of life‐saving treatments [5, 6, 7] mean that even after PPH is detected, women are at continued and unnecessary risk of morbidity and mortality.

Traditionally, PPH treatment has followed a sequential ‘wait‐and‐see’ approach, where healthcare workers monitor the effectiveness of initial treatment before proceeding to the next recommended intervention, if bleeding persists. However, recent evidence has prompted a shift toward using treatment bundles, where all recommended interventions are administered in quick succession or concurrently following PPH detection [8]. This shift was influenced by the findings of the E‐MOTIVE cluster‐randomised trial, which evaluated the effectiveness of Early PPH detection combined with the MOTIVE treatment bundle, across Nigeria, Kenya, Tanzania and South Africa [9]. The MOTIVE bundle included uterine Massage, administration of Oxytocic drugs, Tranexamic acid (TXA), IntraVenous (IV) fluids, Examination of the genital tract, and escalation of treatment as needed, administered in no specific order. The trial demonstrated a 60% reduction in vaginal blood loss ≥ 1000 mL, or laparotomy for bleeding, or maternal death due to bleeding, in the intervention group (E‐MOTIVE care) compared to the control group (usual care).

Consequently, the World Health Organisation (WHO) revised its PPH guidelines to recommend the MOTIVE bundle, with all treatment components to ideally be initiated within 15 min of detection [8]. This recommendation reflects the approach taught to healthcare workers during E‐MOTIVE training. However, a knowledge gap remains regarding how these procedures were implemented in practice during the trial, especially in comparison to usual care practices.

Our research, a nested observational study within the E‐MOTIVE trial, addressed this gap by conducting an in‐depth analysis of PPH treatment processes to provide insights on PPH care. The aim was to gain a more granular understanding of the frequency and timing of PPH treatment initiation in E‐MOTIVE care hospitals compared to usual care hospitals.

## 2 Methods

### 2.1 Study Design

The E‐MOTIVE trial, a multi‐country cluster‐randomised study (NCT04341662), examined early PPH detection and bundled treatment across 78 district, secondary and tertiary level hospitals in Nigeria, Kenya, Tanzania and South Africa [9]. The trial began with a 7‐month baseline phase, during which three additional hospitals in each country participated as adaptive cycle hospitals. These hospitals were used to refine both the intervention (MOTIVE bundle) and the implementation strategies (E‐MOTIVE training, audit and feedback, trolley or carry case, Champions and use of calibrated drape) [10]. Followed by the randomisation of hospitals into either the intervention (39 hospitals adopted E‐MOTIVE care) or control (39 hospitals continued usual care) groups. Following randomisation, a 2‐month transition phase focused on training and integration of the intervention at hospitals allocated to E‐MOTIVE care. The trial concluded with a 7‐month implementation phase during which data on comparative effectiveness were collected.

Healthcare workers in E‐MOTIVE care hospitals received training on regular and timely postpartum maternal assessments, objective quantification of blood loss, as well as specific diagnostic criteria for PPH detection. When a PPH was detected, the MOTIVE treatment bundle was administered, which included uterine Massage, Oxytocic drugs, TXA, IV fluids, genital tract Examination and rapid escalation as required. Healthcare workers were trained to initiate all treatments of the MOTIVE bundle concurrently or in quick succession, and within 15 min of detection. In contrast, healthcare workers at usual care hospitals continued to use subjective measurement of blood loss, relied on local guidelines, which generally advised sequential treatment.

The E‐MOTIVE trial was approved by the relevant ethics and regulatory review committees of each country. Individual‐level consent from women for observations was not obtained, because they were not the target of the E‐MOTIVE intervention, they were not interacted with for data collection, and no identifiable information was recorded. The study adhered to the principles of the Declaration of Helsinki, CIOMS International Ethical Guidelines, and the Ottawa Statement for the Ethical Design and Conduct of Cluster Randomised Trials (Data S1).

Further information regarding the E‐MOTIVE trial [9], and other analyses of this observational study [11, 12] are published elsewhere.

### 2.2 Nested Study Procedures

We observed healthcare workers providing clinical care to women from the moment of vaginal birth until the removal of the obstetric drape during the implementation phase of the trial, conducted between June and December 2022. The observations were conducted using a staggered approach, aligned with the staggered randomisation schedule of the E‐MOTIVE trial for the intervention hospitals, while a pragmatic approach was used for the selection of observation periods in hospitals following usual care. Our observations were direct and passive, capturing consecutive vaginal births across day and night shifts and on both weekdays and weekends, over one‐to‐two weeks at each hospital. This approach was designed to minimise selection bias and capture instances of women experiencing PPH. Through these observations, we aimed to gain an in‐depth understanding of the frequency and timing of PPH treatment initiation in E‐MOTIVE care hospitals compared to usual care hospitals. Specifically, we examined the time from PPH detection to when the first component of the MOTIVE bundle was initiated, the interval from the initiation of the first bundle component to the last bundle component, and a detailed breakdown of each component of the bundle. This nested study did not have independent exclusion criteria; however, women excluded from the primary E‐MOTIVE trial were also excluded from the nested study by default.

We utilised two structured observation guides: one tailored for hospitals implementing E‐MOTIVE care and another for those following usual care (Data S2). The key difference between the guides was the inclusion of procedures using the calibrated drape to facilitate early PPH detection in E‐MOTIVE care hospitals. The usual care hospital used an uncalibrated drape to measure blood loss, which served as the primary study outcome component in both groups. These hospitals did not receive any additional clinical training beyond what was provided routinely in their facilities. There were no differences between the guides regarding treatment administration or timing. Both guides document patient characteristics, time of birth, drape funnel opening and removal, active management of the third stage and postpartum maternal assessments. For PPHs detected, the time of detection, diagnostic methods, blood loss thresholds for detection, treatments and doses administered, and escalation of care procedures were recorded, along with start and stop timestamps. Additionally, medication doses and the healthcare worker cadre responsible for each aspect of care were documented. To avoid duplication of data entry, the following data were sourced from the primary E‐MOTIVE trial database for the observed women: maternal age, gestational age at birth, pregnancy type, number of previous births ≥ 24 weeks, history of caesarean section, previous PPH, episiotomy, perineal tear, laparotomy for bleeding, blood transfusion, intensive care admission, and final blood loss quantification based on the verified drape weight.

Observations were carried out by a team of implementation and research midwives employed by the E‐MOTIVE trial, all clinically trained and knowledgeable about the hospital's PPH protocols and guidelines. Training for the 148 observers was conducted through 17 Zoom sessions. Observations were initially recorded on a paper guide before being entered into REDCap on the same day. Data entries were reviewed daily to ensure accuracy, maintain clinical coherence and resolve any missing information.

### 2.3 Outcomes

The population of interest comprised all women who gave birth vaginally at the 78 hospitals participating in the E‐MOTIVE trial and had a PPH clinically detected and treated by a healthcare worker, irrespective of the final objective blood loss measurement after the obstetric drape was removed.

We compared the management of PPH (uterine Massage, Oxytocics, TXA, IV fluids and genital tract Examination) between E‐MOTIVE care hospitals and usual care hospitals, focusing on three key aspects, both for individual treatment components and as the MOTIVE bundle:

1. The duration from the initiation of the first to the last treatment component of the MOTIVE bundle, when all components were administered.
2. The frequency of PPH treatments administered.
3. The timing from PPH detection to treatment initiation.

### 2.4 Analysis

During the implementation phase of the E‐MOTIVE trial, a subgroup representing approximately 5% of vaginal births (an estimated 4,983 out of 99,659 women) was selected for observation. Given an expected PPH prevalence of up to 10% [13], we anticipated approximately 498 women would experience PPH among the expected 4983 observations. The required number of observations was estimated using historical data from each participating hospital, with the data collection period tailored to fit within the trial's established timeframe.

We conducted a descriptive analysis stratified by E‐MOTIVE care and usual care using STATA 18. We reported frequency and percentages for categorical data, and median and interquartile range (IQR) for continuous measures, due to skewness of the data. All analyses used complete case analysis with minimal missing data.

## 3 Results

**FIGURE 1:**
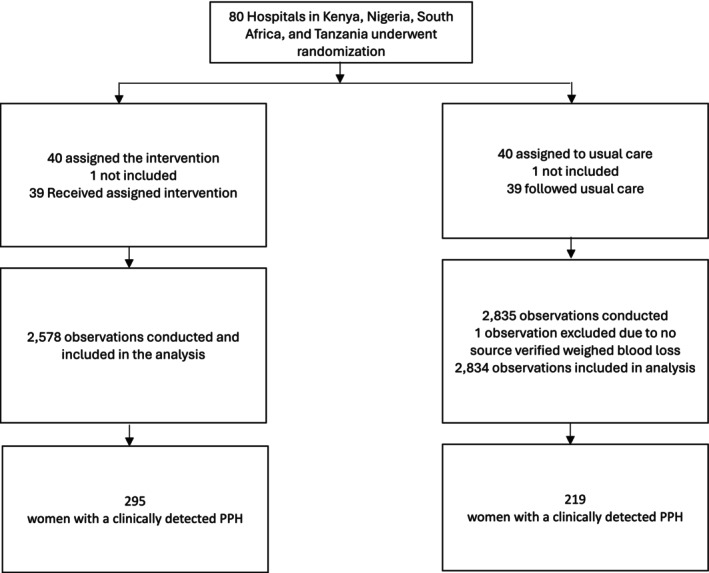
Consort diagram.

This nested study involved 5413 direct observations of clinical care performed by healthcare workers, with a distribution of 2578 observations at 39 hospitals implementing E‐MOTIVE care and 2835 at 39 hospitals following usual care in Nigeria, Kenya, Tanzania and South Africa. We excluded one observation from usual care, as it was excluded from the E‐MOTIVE trial due to missing source verified drape weight (Figure 1). This left 5412 observations available for analysis. Among these observations, 514 women with PPH were recorded—295 in E‐MOTIVE care and 219 in usual care. Postpartum haemorrhage was determined based on clinical detection by a healthcare worker independent of the final blood loss quantification after the drape was removed.

Overall, the baseline characteristics of women with PPHs in both the E‐MOTIVE care and usual care hospitals showed a high degree of similarity, although there were some notable differences. Haemoglobin was tested less often in the E‐MOTIVE care group (51.2%, 151/295) compared to the usual care group (74%, 162/219). Also, fewer women took iron tablets for more than 1 month in the E‐MOTIVE care group (58%, 171/295) compared to the usual care group (68%, 149/219). Augmentation of labour was less frequent in the E‐MOTIVE care group (7.1%, 21/295) compared to the usual care group (18.3%, 40/219). There were more women with an episiotomy in the E‐MOTIVE care group than the usual care group (20.3%, 60/295 vs. 15.1%, 33/219) and fewer women with a vaginal tear (33.6%, 99/295 vs. 40.2%, 88/219) (Table 1). For active management of the third stage of labour, misoprostol was administered more often in the E‐MOTIVE care group (40%, 118/295), compared to the usual care group (22.8%, 50/219). Furthermore, the placenta was checked for completeness of the membranes and cotyledons less often in the E‐MOTIVE care group (60%, 177/295) compared to the usual care group (94.5%, 207/219). The full table of baseline characteristics is listed in Data S3. The subset of women in this nested study is representative of the larger trial population. All baseline characteristics are detailed in Table S1.

**TABLE 1:** Baseline characteristics.

Baseline characteristics	E‐MOTIVE care	Usual care
	*N* = 295	*N* = 219
**Pregnancy information**		
Maternal age, median [IQR]	25 [21–30]	24 [20–30]
Gestational age at birth, median [IQR]	38 [37–40]	39 [38–40]
Previous caesarean section, (*n*/*N*, %)	12 (4.1)	9 (4.1)
Previous postpartum haemorrhage, (*n*/*N*, %)	16 (5.4)	11 (5)
**Type of pregnancy**		
Singleton, (*n*/*N*, %)	276 (93.6)	215 (98.2)
Twin, (*n*/*N*, %)	19 (6.4)	4 (1.8)
**Health conditions**		
Body mass index, median [IQR]	26.42 [22.86–31.25]	26.03 [23.05–29.09]
Hypertension, frequency, (*n*/*N*, %)	12 (4.1)	10 (4.6)
Malaria, (*n*/*N*, %)	3 (1)	12 (5.5)
Uterine fibroids, (*n*/*N*, %)	0 (0)	2 (0.9)
**Pregnancy, labour, birth risk factors**		
Frequency of haemoglobin testing in pregnancy, (*n*/*N*, %)	151 (51.2)	162 (74)
Haemoglobin levels, median[IQR]	112 [102–120]	117 [105–128]
Taking iron tablets for > 1 month in pregnancy, (*n*/*N*, %)	171 (58)	149 (68)
Placenta previa or low lying, accreta, increta or percreta, (*n*/*N*, %)	1 (0.3)	0 (0)
Placental abruption, (*n*/*N*, %)	7 (2.4)	6 (2.7)
Pregnancy induced hypertension, (*n*/*N*, %)	22 (7.5)	11 (5)
Pre‐eclampsia, (*n*/*N*, %)	16 (5.4)	12 (5.5)
Eclampsia, (*n*/*N*, %)	4 (1.4)	1 (0.5)
Antepartum haemorrhage, (*n*/*N*, %)	5 (1.7)	12 (5.5)
Intrapartum haemorrhage, (*n*/*N*, %)	0 (0)	4 (1.8)
Pushing > 60 min, (*n*/*N*, %)	8 (2.7)	13 (5.9)
Induction of labour, (*n*/*N*, %)	21 (7.1)	22 (10.1)
Augmentation of labour, (*n*/*N*, %)	21 (7.1)	40 (18.3)
Episiotomy, (*n*/*N*, %)	60 (20.3)	33 (15.1)
Vaginal/Perineal tear, (*n*/*N*, %)	99 (33.6)	88 (40.2)
**AMTSL: Medicines administered**		
Oxytocin, (*n*/*N*, %)	291 (98.6)	218 (99.5)
Misoprostol, (*n*/*N*, %)	118 (40)	50 (22.8)
**AMTSL: Management of the placenta**		
CCT performed, (*n*/*N*, %)	282 (95.6)	208 (95)
MROP performed, (*n*/*N*, %)	13 (4.4)	11 (5)
Placenta checked, (*n*/*N*, %)	177 (60)	207 (94.5)

Abbreviations: % = percentage (*n*/*N*); AMTSL = Active management of third stage of labour; IQR = interquartile range; *n* = number; *N* = total number.

**FIGURE 2:**
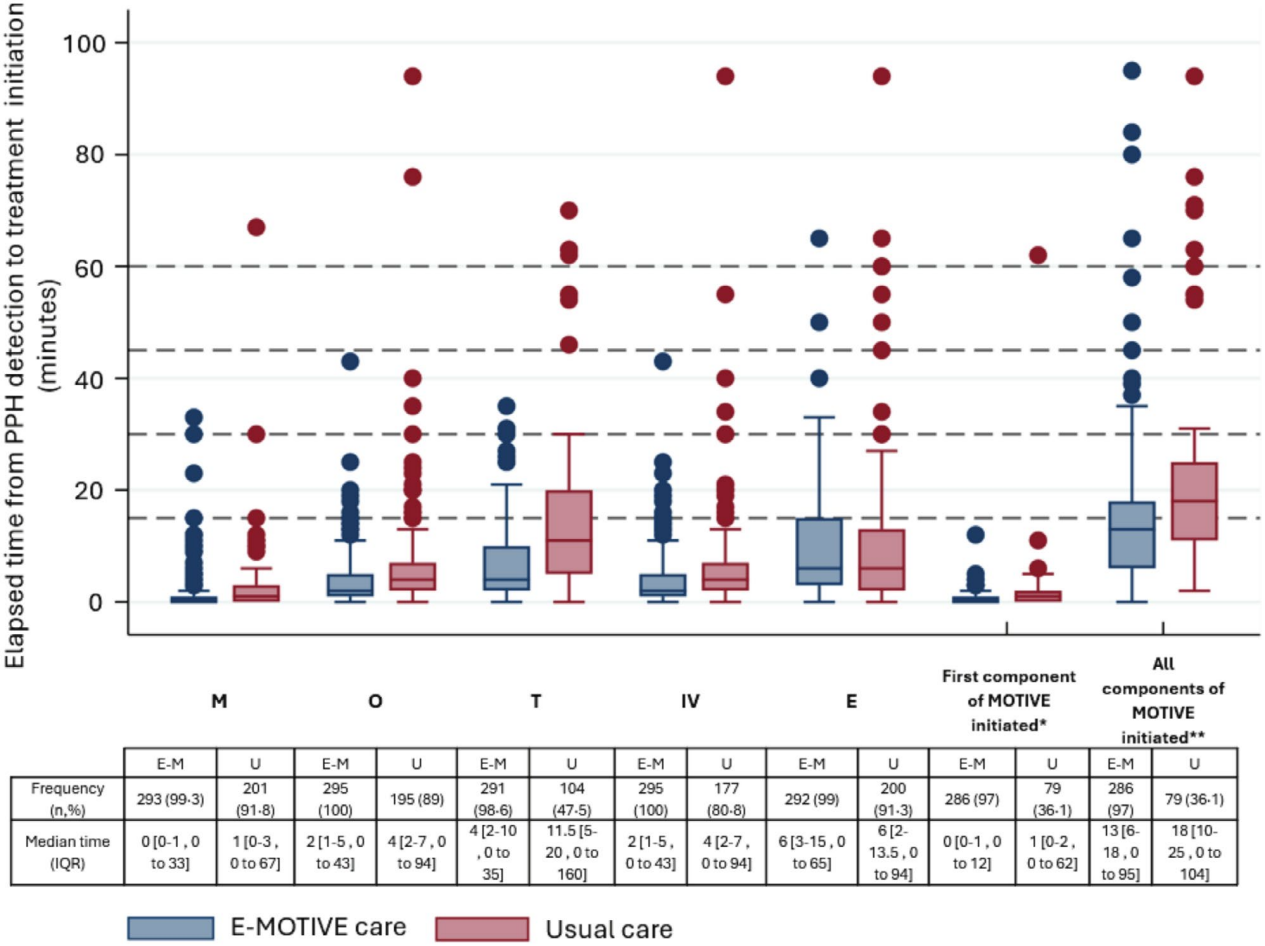
Elapsed time from postpartum haemorrhage detection to each treatment and MOTIVE bundle initiated. M = uterine massage, O = Oxytocics; T = tranexamic acid; IV = intravenous fluids; E = examination of the genital tract; First component of MOTIVE initiated * = Elapsed time from PPH detection to first treatment initiated when ALL MOTIVE components administered; All components of MOTIVE initiated ** = Elapsed time from first treatment initiated to last treatment initiated when all the MOTIVE bundle components administered; Excluded from boxplot are the following outliers in usual care: 2 for tranexamic acid initiated, 2 for last treatment initiated. The dotted lines represent the 15 min intervals until 60 min postpartum.

The MOTIVE bundle was administered to significantly more women with PPH in the E‐MOTIVE care group than in usual care, with 96.9% (286/295) of women with PPH receiving all components in the E‐MOTIVE care group, compared to only 36.1% (79/219) in the usual care group. The median time to initiate the first component of the bundle was similar between the groups (E‐MOTIVE care: 0 min, IQR 0–1; usual care: 1 min, IQR 0–2). However, E‐MOTIVE care demonstrated a much narrower distribution, with a maximum initiation time of 12 min, compared to 62 min in usual care hospitals (Figure 2 and Table 2).

**TABLE 2:** Elapsed time from clinical postpartum haemorrhage detection to treatment(s) initiated.

Treatment components initiated	Frequency (*n*, %)		Elapsed time from clinical PPH detection to treatment initiation							
			≤ 15 min (*n*, %)		16–20 min (*n*, %)		≥ 21 min (*n*, %)		Median time in minutes (IQR)	
	E‐M	U	E‐M	U	E‐M	U	E‐M	U	E‐M	U
	*N* = 295	*N* = 219	*N* = 295	*N* = 219	*N* = 295	*N* = 219	*N* = 295	*N* = 219	*N* = 295	*N* = 219
Uterine massage	293 (99.3)	201 (91.8)	290 (98.3)	199 (90.9)	0 (0)	0 (0)	3 (1)	2 (0.9)	0 [0–1, 0–33]	1 [0–3, 0–67]
Oxytocic	295 (100)	195 (89)	289 (98)	176 (80.4)	4 (1.4)	9 (4.1)	2 (0.7)	10 (4.6)	2 [1–5, 0–43]	4 [2–7, 0–94]
Oxytocin	295 (100)	180 (82.2)	288 (97.6)	166 (75.8)	4 (1.4)	6 (2.7)	3 (1)	8 (3.7)	3 [1–5, 0–43]	4 [2–7, 0–94]
Misoprostol	58 (19.7)	83 (37.9)	38 (12.9)	49 (22.4)	5 (1.7)	12 (5.5)	15 (5.1)	22 (10)	7 [3–21, 0–95]	14 [5–22, 0–119]
Syntometrine	2 (0.7)	0 (0)	0 (0)	0 (0)	0 (0)	0 (0)	2 (0.7)	0 (0)	28.5 [22–35, 22–35]	0 [0–0, 0–0]
TXA	291 (98.6)	104 (47.5)	268 (90.9)	64 (29.2)	12 (4.1)	16 (7.3)	11 (3.7)	24 (11)	4 [2–10, 0–35]	11.5 [5–20, 0–160]
IV fluids	295 (100)	177 (80.8)	288 (97.6)	163 (74.4)	4 (1.4)	6 (2.7)	3 (1)	8 (3.7)	2 [1–5, 0–43]	4 [2–7, 0–94]
Examination‐genital tract	292 (99)	200 (91.3)	229 (77.6)	165 (75.3)	32 (10.9)	12 (5.5)	31 (10.5)	23 (10.5)	6 [3–15, 0–65]	6 [2–13.5, 0–94]
Suturing*	144/292 (48.8)	113/200 (51.6)	75 (25.4)	63 (28.8)	14 (4.8)	13 (5.9)	55 (18.6)	37 (17)	15 [9–27.5, 0–835]	14 [9–25, 0–100]
Escalation	29 (9.8)	9 (4.1)	3 (1)	4 (1.8)	5 (1.7)	0 (0)	21 (7.1)	5 (2.3)	30 [19–46, 0–314]	21 [7–43, 0–49]
In cases where all WHO MOTIVE components** were administered: Time from PPH detection to first treatment initiation	286 (97)	79 (36.1)	286 (97)	78 (35.6)	0 (0)	0 (0)	0 (0)	1 (0.5)	0 [0–1, 0–12]	1 [0–2, 0–62]
In cases where all WHO MOTIVE components** were administered: Time from 1st to *last* treatment initiation	286 (97)	79 (36.1)	191 (64.8)	37 (16.9)	42 (14.2)	11 (5)	53 (18)	31 (14.2)	13 [6–18, 0 to 95]	18 [10–25, 0 to 104]

*Note:* *Denominator for suturing is the total number of women who had an examination of the genital tracts for PPH management: E‐MOTIVE = 292, Usual care = 200; All WHO MOTIVE components ** = uterine massage, oxytocics, tranexamic acid, intravenous fluids and examination of the genital tract.

Abbreviations: % = percentage; E‐M = E‐MOTIVE care; U = usual care; IQR = interquartile range; IV = intravenous; *n* = number; N = total number; PPH = postpartum haemorrhage; TXA = tranexamic acid.

The duration between initiation of the first and the last MOTIVE components was shorter in the E‐MOTIVE care group, with a median time of 13 min (IQR 6–18), compared to 18 min (IQR 10–25) in the usual care group. Additionally, 64.8% (191/295) of women with PPH in the E‐MOTIVE care group had all MOTIVE components initiated within 15 min of detection, compared to only 16.9% (37/219) in the usual care group. In total, 78.9% (233/295) of women in the E‐MOTIVE care group had all components initiated within 20 min of PPH detection, compared to 21.9% (48/219) in the usual care group.

Uterine massage was performed for most women with PPH in both care groups (E‐MOTIVE care: 99.3%, 293/295; usual care: 91.8%, 201/295). The median time to initiate uterine massage was similar between the groups (E‐MOTIVE care: 0 min, IQR 0–1; usual care: 1 min, IQR 0–3). However, the maximum time to initiate uterine massage differed significantly: in E‐MOTIVE care hospitals, the maximum time was 33 min, while in usual care hospitals, it was 67 min after PPH detection (Figure 2 and Table 2).

An oxytocic drug was administered to all women with PPH in the E‐MOTIVE care group (100%, 295/295), compared to 89% (195/219) in the usual care group. The median time to administer the first oxytocic drug after PPH detection was slightly shorter in the E‐MOTIVE care group (2 min, IQR 1–5) than in the usual care group (4 min, IQR 2–7). The maximum time to initiate oxytocic treatment differed significantly, with a maximum of 43 min in the E‐MOTIVE care group compared to up to 94 min in the usual care group (Figure 2 and Table 2). The administration of individual oxytocics—oxytocin, misoprostol and syntometrine—was analysed separately, with further details provided in Table 2.

Tranexamic acid was administered to considerably more women with PPH in the E‐MOTIVE care group (98.6%, 291/295) compared to the usual care group (47.5%, 104/219). The median time to administer TXA was much quicker in the E‐MOTIVE care group (4 min, IQR 2–10) than in the usual care group (11.5 min, IQR 5–20). The maximum time to initiate TXA treatment was notably shorter in the E‐MOTIVE care group, with a maximum of 35 min, compared to 160 min in the usual care group (Figure 2 and Table 2).

All women with PPH in the E‐MOTIVE care group received IV fluids (100%, 295/295), compared to 80.8% (171/219) of women in the usual care group. IV fluids were initiated slightly quicker in the E‐MOTIVE care group, with a narrower distribution (median time: 2 min, IQR: 1–5), compared to the usual care group (median time: 4 min, IQR: 2–7). The maximum time to initiation was also much quicker in the E‐MOTIVE care group (43 min) compared to the usual care group (94 min) (Figure 2 and Table 2).

Most women in both care groups received a genital tract examination (E‐MOTIVE care: 99%, 292/295; usual care: 91.3%, 200/219), with median time to examination of 6 min after PPH detection in both groups (E‐MOTIVE care IQR: 3–15 min; usual care IQR: 2–13.5 min) (Figure 2 and Table 2).

## 4 Discussion

### 4.1 Main Findings

In E‐MOTIVE care hospitals, women received timelier and more comprehensive PPH treatment through the MOTIVE bundle. Adherence to the bundle was exceptionally high, with 97% of women receiving all components after healthcare workers completed E‐MOTIVE training. Detailed timing data showed that all MOTIVE bundle components were initiated as quickly as 6 min, with a median initiation time of 13 min. Furthermore, 79% of these women had all components administered within 20 min of PPH detection. Additionally, the introduction of the PPH trolley or carry case, as one of the accepted implementation strategies [11] provided all necessary items in a readily available and easily accessible manner. This may have facilitated implementation and contributed to the observed higher frequency and quicker initiation of the MOTIVE bundle in the E‐MOTIVE care group. Women in E‐MOTIVE care hospitals received more frequent and timelier PPH detection and accelerated PPH treatment which together help understand the clinical care processes that contributed to the improved outcomes observed in the E‐MOTIVE trial.

### 4.2 Strengths and Limitations

The main strength of this study is its integration within the larger E‐MOTIVE cluster‐randomised trial, allowing a comparison of practices across diverse healthcare environments in four LMIC countries. Observers with clinical backgrounds employed by the trial, and already familiar with the clinical settings and routine staff, conducted the observations. This arrangement helped minimise disruptions and reduce potential ‘Hawthorne effects’ that might otherwise alter healthcare worker behaviour [14]. Evidence suggests that staff tend to resume typical routines over time, which supports more natural interactions during prolonged observations [15, 16]. A limitation of this study is that, despite more than 5400 observations conducted across both E‐MOTIVE care and usual care hospitals, the final number of 514 detected PPHs were relatively small, with 295 (11%) detected PPHs in E‐MOTIVE care and 219 (8%) in usual care. Despite this, the number of observed PPHs which were diagnosed was slightly higher than expected, with an total number of 514 compared to an expected 498. Future research should aim to include larger sample sizes across a broader range of LMICs. This will help strengthen the evidence on the timing of PPH treatment initiation and improve the generalisability of findings to inform healthcare practices on a larger scale.

### 4.3 Interpretation

Our study demonstrated that the implementation of the MOTIVE bundle improved PPH treatment initiation in the E‐MOTIVE care group by enabling more consistent application of the bundle and quicker initiation of treatments compared to the usual care group. The findings highlight the achievement of rapid treatment initiation in the intervention hospitals, with all MOTIVE components initiated within 15 min of PPH detection and a target of no more than 20 min for full initiation. These improvements in treatment timing and consistency are critical, as delays in care are a major contributor to maternal morbidity and mortality in LMICs. The observed differences in outcomes between the E‐MOTIVE care group and usual care group could reflect a combination of earlier PPH detection, the integration of implementation strategies [11], and comprehensive, timely treatment initiation of the MOTIVE bundle. These elements likely worked together to reduce delays in care. Given these findings, the scale‐up of E‐MOTIVE care should not only prioritise the timely initiation of the MOTIVE bundle but also address systemic barriers to rapid PPH management to optimise impact. This study provides valuable insights into the positive results of the E‐MOTIVE trial, which can be attributed to its structured, protocol‐driven approach, which ensured timely, comprehensive, and coordinated PPH treatment initiation using the MOTIVE bundle. This approach is particularly important in LMIC settings, where delayed or fragmented care often contributes to adverse outcomes due to PPH.

Numerous studies have highlighted significant variations and delays in the administration of PPH treatments in LMICs, contributing to poor maternal outcomes related to PPH. A nationwide cross‐sectional study in Nigeria highlighted these concerns, revealing significant delays in PPH treatment, with 25% of women who died from PPH experiencing delays of at least 4 h before treatment was initiated after PPH was detected [7]. Although the outcomes in the usual care hospitals were less favourable than in E‐MOTIVE care hospitals, they still outperformed those in several other studies. For instance, Ansari et al. [5], reported substantial discrepancies in the administration of key treatments, with TXA being administered to only 6% of women with PPH. Oxytocin was administered in 50% of women with PPH, and misoprostol in 22%, while uterine massage and IV fluids were more frequently administered in 88% and 90% of PPHs, respectively. Similarly, Clarke‐Deelder et al. [6], found that only 23% of women received all recommended treatments. These comparisons emphasise the urgent need for standardised, evidence‐based protocols such as E‐MOTIVE to bridge critical gaps in PPH management across diverse healthcare settings.

### 4.4 Conclusion

Our study is the first to directly observe the timing of the MOTIVE bundle initiation and its individual components following PPH detection, nested within the large, multi‐country, randomised‐control E‐MOTIVE Trial. Our findings highlight that timely and comprehensive PPH management using the MOTIVE bundle (uterine Massage, Oxytocic drugs, Tranexamic acid, IV fluids and Examination of the genital tract) was achieved in the E‐MOTIVE care hospitals, with initiation of all components within 15–20 min of PPH detection in most instances.

### 4.5 Practical Recommendations

Timely and coordinated approach is important for improving PPH management, particularly in low‐ and middle‐income countries where PPH‐related adverse outcomes are most prevalent. Integrating these findings into clinical guidelines, clinical education, and ongoing training and scale‐up will ensure timely PPH management, improving maternal health outcomes.

## Author Contributions

K‐M.M., I.G., A.C. conceptualised and designed this study. K‐M.M., A.L.F., R.Y., F.Ab., L.C.A., J.K.M., P.O., M.S., A.B., F.S., S.W. and S.D.K. were involved in data curation and K‐M.M., A.L.F., R.Y., F.Ab., L.C.A., H.M.S., A.A.W., J.O., A.M., M.S‐M., E.A., E.M. and A.D. were involved in project administration. K‐M.M., H.G., Z.Q., F.A.A.‐B., M.S‐M., S.F. and N.M. supervised the study implementation. K‐M.M., F.Al., C.E. and J.M. conducted data analysis and visualisation. K‐M.M. wrote the original draft. All authors contributed to the interpretation of results and revised the manuscript. K‐M.M. and A.C. accept responsibility for the decision to submit for publication.

## Ethics Statement

The E‐MOTIVE trial was approved by the relevant ethics and regulatory review committees of each country between March and June 2020. Individual‐level consent from women for observations was not obtained, because they were not the target of the E‐MOTIVE intervention, they were not interacted with for data collection, and no identifiable information was recorded. The study adhered to the principles of the Declaration of Helsinki, CIOMS International Ethical Guidelines, and the Ottawa Statement for the Ethical Design and Conduct of Cluster Randomised Trials (Data S1).

## Conflicts of Interest

Justus Hofmeyr has conceived a re‐usable device for postpartum blood loss monitoring, the Maternawell Tray, which is marketed by Maternova, a global women's health solutions company that holds the intellectual property. JH benefited from consulting fees in the past as inventor but receives no current income nor prospect of future income from this project. The remaining authors declare that they have no competing interests.

## Supporting information


**Data S1.** Ethics approvals and permissions.
**Data S2.** Observation guides.
**Table S1.** All baseline characteristics.

## Data Availability

Study protocol, study instruments, and de‐identified observation data and statistical code underlying the results reported in this article will be made available after de‐identification, upon request to the corresponding author immediately following publication until 5 years following publication. A data sharing agreement will require a commitment to using the data only for specified research purposes, to researchers who provide a methodologically sound proposal, to securing the data appropriately, and to destroying the data after a nominated period.
