# Supplementary material for: When Is Postpartum Haemorrhage Treatment Initiated? A Nested Observational Study Within the E‐MOTIVE Trial
Source: BJOG. 2025 Aug 4;132(11):1664–72. doi: 10.1111/1471-0528.18293 (PMC12411655; doi:10.1111/1471-0528.18293)
Supplement: Supplementary file 1 — Data S1. Ethics approvals and permissions. Data S2. Observation guides. Table S1. All baseline characteristics. [file BJO-132-1664-s001.docx]

**Supplementary material S1 – Ethics approvals and permissions**

This study has received ethics approval and permissions from the following entities:

**United Kingdom**:

University of Birmingham STEM committee (Reference number: ERN_19-1557E).

**Kenya**:

University of Nairobi: KNH/ERC/Mod&SAE/430;

Pharmacy and Poisons Board PPB/ECCT/20/06/06/2020(116),

National Commission for Science, Technology and Innovation Nacosti P/21/8437.

**Nigeria**:

National Health Research Ethics Committee of Nigeria (NHREC) (Reference number: NHREC/01/01/2007)-07/04/2022D).

**South Africa**:

Eastern Cape Department of Health (EC_202007_015);

University of Cape Town Human Research Ethics Committee (HREC; reference number: 091/2020),

Health Province of KwaZulu-Natal (NHRD reference number: KZ_202008_036),

University of the Witwatersrand Human Research Ethics Committee-Medical (reference number: M200241).

**Tanzania**:

Muhimbili University of Health and Allied Sciences (MUHAS) (reference number: DA.282/298/06/C/767);

National Institute for Medical Research (NIMR) (Reference number: NIMR/HQ/R.8a/Vol IX/3510).

The E-MOTIVE trial aimed to change health worker behaviours regarding PPH detection and management. Hospital leadership at each site provided facility-level permission for the observations, while healthcare providers gave written informed consent before trial-specific training. Monitoring visits and observations of healthcare professionals managing vaginal births were conducted during baseline and intervention phases. Individual-level consent from women for these observations was not sought, as women were not the target of the intervention, were not interacted with for observation data collection, and no identifiable information on the observed women was collected or linked to them. The trial was conducted in accordance with the Declaration of Helsinki, CIOMS International Ethical Guidelines, and the Ottawa Statement for the Ethical Design and Conduct of Cluster Randomised Trials.

**Supplementary material S2- Observation guides**

E-MOTIVE care - <https://osf.io/7xmgc>

Usual care - <https://osf.io/m47pt>

**Supplementary Table**

Table S1: All baseline characteristics

| **Characteristics** | **E-MOTIVE care** | **Usual care** |
| --- | --- | --- |
|  | **N=295** | **N=219** |
| **Pregnancy information** |  |  |
| Maternal age |  |  |
| mean (SD) | 26·1 (6·2) | 25·6 (6·5) |
| median[IQR] | 25 [21-30] | 24 [20-30] |
| Gestational age at birth |  |  |
| mean (SD) | 38·2 (2.6) | 38·4 (2·1) |
| median[IQR] | 38 [37-40] | 39 [38-40] |
| UNKNOWN gestational age, no./total no. (%) | 22 (7·5) | 4 (1·8) |
| Type of pregnancy |  |  |
| Singleton, no./total no. (%) | 276 (93·6) | 215 (98·2) |
| Twin, no./total no. (%) | 19 (6·4) | 4 (1·8) |
| Number of previous births ≥ 24 weeks gestation, no./total no. (%) | 290 (98·3) | 216 (98·6) |
| UNKNOWN Number of previous births ≥ 24 weeks, no./total no. (%) | 5 (1·7) | 3 (1·4) |
| Previous caesarean section, no./total no. (%) | 12 (4·1) | 9 (4·1) |
| Previous postpartum haemorrhage, no./total no. (%) | 16 (5·4) | 11 (5) |
| **Health conditions** |  |  |
| Body Mass Index |  |  |
| mean (SD) | 27·3 (5·6) | 26·4 (4·6) |
| median[IQR] | 26·4 [22·9-31·3] | 26 [23·1-29·1] |
| < 18.5 no./total no. (%) | 4 (1·4) | 4 (1·8) |
| 18.5 – 24.9 no./total no. (%) | 52 (17·6) | 58 (26·5) |
| ≥ 25 no./total no. (%) | 90 (30·5) | 97 (44·3) |
| Hypertension, frequency, no./total no. (%) | 12 (4·1) | 10 (4·6) |
| Diabetes, frequency, no./total no. (%) | 1 (0·3) | 0 (0) |
| Autoimmune disease, no./total no. (%) | 1 (0·3) | 0 (0) |
| Sexually transmitted infection, no./total no. (%)) | 5 (1·7) | 1 (0·5) |
| Kidney disease, no./total no. (%) | 1 (0·3) | 0 (0) |
| Human Immunodeficiency Virus, no./total no. (%) | 12 (4·1) | 3 (1·4) |
| Malaria, no./total no. (%) | 3 (1) | 12 (5·5) |
| Hepatitis B, no./total no. (%) | 0 (0) | 3 (1·4) |
| Uterine fibroids, no./total no. (%) | 0 (0) | 2 (0·9) |
| **Other maternal health conditions, free text specified** |  |  |
| Unbooked, no./total no. (%) | 14 (4·8) | 6 (2·7) |
| Pyelonephritis, no./total no. (%) | 1 (0·3) | 0 (0) |
| Urinary tract infection, no./total no. (%) | 0 (0) | 1 (0·5) |
| **Pregnancy, labour, birth risk factors** |  |  |
| Haemoglobin tested in pregnancy, no./total no. (%) | 151 (51·2) | 162 (74) |
| Haemoglobin |  |  |
| mean (SD) | 110·1 (16·7) | 116·1 (18·5) |
| median[IQR] | 112 [102-120] | 117 [105-128] |
| Taking iron tablets for > 1 month in pregnancy,  no./total no. (%) | 171 (58) | 149 (68) |
| Previous neonatal death, no./total no. (%) | 13 (4·4) | 10 (4·6) |
| Hookworm treated in pregnancy, no./total no. (%) | 34 (11·5) | 60 (27·4) |
| Placenta previa or low lying, accreta, increta or percreta, no./total no. (%) | 1 (0·3) | 0 (0) |
| Intrauterine growth restriction, no./total no. (%) | 0 (0) | 1 (0·5) |
| Polyhydramnios, no./total no. (%) | 1 (0·3) | 0 (0) |
| Oligohydramnios, no./total no. (%) | 0 (0) | 1 (0·5) |
| Placental abruption, no./total no. (%) | 7 (2·4) | 6 (2·7) |
| Chorioamnionitis, no./total no. (%) | 1 (0·3) | 0 (0) |
| Pregnancy induced hypertension, no./total no. (%) | 22 (7·5) | 11 (5) |
| Pre-eclampsia, no./total no. (%) | 16 (5·4) | 12 (5·5) |
| Eclampsia, no./total no. (%) | 4 (1·4) | 1 (0·5) |
| Antepartum haemorrhage, no./total no. (%) | 5 (1·7) | 12 (5·5) |
| Intrapartum haemorrhage, no./total no. (%) | 0 (0) | 4 (1·8) |
| Febrile in labour (temperature >38°C),  no./total no. (%) | 2 (0·7) | 0 (0) |
| Pushing > 60 minutes, no./total no. (%) | 8 (2·7) | 13 (5·9) |
| Induction of labour, no./total no. (%) | 21 (7·1) | 22 (10·2) |
| Augmentation of labour, no./total no. (%) | 21 (7·1) | 40 (18·3) |
| Received antibiotics in labour, no./total no. (%) | 9 (3·1) | 7 (3·2) |
| Meconium liquor, no./total no. (%)) | 12 (4·1) | 6 (2·7) |
| Compound presentation, no./total no. (%) | 0 (0) | 6 (2·7) |
| Breech presentation, no./total no. (%) | 0 (0) | 3 (1·4) |
| Malpresentation or malposition, no./total no. (%) | 1 (0·3) | 1 (0·5) |
| Episiotomy, no./total no. (%) | 60 (20·3) | 33 (15·1) |
| Vaginal/Perineal tear, no./total no. (%) | 99 (33·6) | 88 (40·2) |
| Shoulder dystocia, no./total no. (%) | 4 (1·4) | 1 (0·5) |
| Intrauterine fetal death, no./total no. (%) | 1 (0·3) | 0 (0) |
| Obstructed labour, no./total no. (%) | 1 (0·3) | 0 (0) |
| Semi conscious, no./total no. (%) | 0 (0) | 1 (0·5) |
| Baby 1 - mode of birth |  |  |
| Spontaneous vaginal, no./total no. (%) | 293 (99·3) | 217 (99·1) |
| Ventouse, no./total no. (%) | 2 (0·7) | 2 (0·9) |
| Baby 2 - mode of birth |  |  |
| Spontaneous vaginal, no./total no. (%) | 19 (6·4) | 4 (1·8) |
| **AMTSL: Medicines administered** |  |  |
| Oxytocin, (n/N, %) | 291 (98.6) | 218 (99·5) |
| Misoprostol, (n/N, %) | 118 (40) | 50 (22·8) |
| **AMTSL: Management of the placenta** |  |  |
| CCT performed, (n/N, %) | 282 ( 95·6) | 208 (95) |
| MROP performed, (n/N, %) | 13 (4·4) | 11 (5) |
| Placenta checked, (n/N, %) | 177 (60) | 207 (94·5) |

SD = standard deviation; IQR = interquartile range; n = number; % = percentage; n and no. = number; AMTSL= Active management of third stage of labour
